# Patients’ knowledge, attitudes, and practices concerning endometriosis and its long-term management

**DOI:** 10.1186/s12905-025-04187-z

**Published:** 2025-11-28

**Authors:** Chengbin Lin, Zhiwei Wu, Yuanjun Cai

**Affiliations:** Department of Obstetrics and Gynecology, College of Clinical Medicine for Obstetrics & Gynecology and Pediatrics, Fujian Maternity and Child Health Hospital, Fujian Medical University, No.18, Daoshan Road, Fuzhou, Fujian 350000 China

**Keywords:** Knowledge, Attitude, Practice, Endometriosis, Management, Cross-sectional study

## Abstract

**Background:**

Endometriosis, affecting 10–15% of women, causes pain and infertility. Current treatments have limitations. This study aimed to investigate patients’ knowledge, attitude, and practice (KAP) concerning endometriosis and its long-term management.

**Methods:**

A cross-sectional study was conducted among patients diagnosed with endometriosis, using a self-designed questionnaire.

**Results:**

A total of 301 valid questionnaires were included. The mean age of the participants was 32.3 ± 6.8 years, and 220 (73.1%) cases or participants. The participants scored a mean of 13.28 ± 5.98 (possible range: 0–24) for knowledge, 28.23 ± 2.38 (possible range: 8–40) for attitude, and 22.56 ± 4.76 (possible range: 7–35) for practice. They indicated a generally poor KAP. The structural equation model revealed that knowledge had a direct influence on attitude (β = 0.287, *P* = 0.014) and knowledge also directly influenced practice (β = 0.361, *P* = 0.005). Duration of endometriosis (β = 0.300, *P* = 0.006), having children (β = -0.127, *P* = 0.010), ethnicity (β = -0.127, *P* = 0.010), education (β = 0.217, *P* = 0.009), and having female relatives with endometriosis (β = -0.202, *P* = 0.004) had significant direct effects on knowledge. Having children (β = -0.037, *P* = 0.015), ethnicity (β = -0.037, *P* = 0.008), having female relatives with endometriosis (β = -0.058, *P* = 0.004), income (β = -0.176, *P* = 0.009), drinking (β = 0.318, *P* = 0.008), and health insurance (β = 0.169, *P* = 0.016) influenced attitude. Duration of endometriosis diagnosis (β = 0.103, *P* = 0.006), having children (β = -0.044, *P* = 0.010), ethnicity (β = -0.242, *P* = 0.015), education (β = 0.258, *P* = 0.013), having female relatives with endometriosis (β = -0.293, *P* = 0.007), and health insurance (β = -0.123, *P* = 0.030) influenced practice.

**Conclusion:**

Patients demonstrated inadequate knowledge, moderate attitudes, and practices regarding endometriosis. These findings suggest a need for more targeted educational efforts. Healthcare providers should prioritize education, particularly for individuals from diverse backgrounds and those with limited resources, to enhance patient outcomes.

**Supplementary Information:**

The online version contains supplementary material available at 10.1186/s12905-025-04187-z.

## Background

Endometriosis, a common gynecological condition affecting women of reproductive age, is the second most prevalent gynecological disease after uterine fibroids [1, 2]. It is an estrogen-dependent, chronic, non-malignant, inflammatory disease defined by the presence of endometrial-like glands and stroma outside the uterine cavity [3–5]. This multifaceted condition is characterized by classic symptoms of pain, infertility, and a debilitating impact on quality of life [6] and has an estimated prevalence of approximately 10%−15% in the general population [7, 8]. The peak incidence of endometriosis typically occurs around the age of 30 [9]. The development of endometriosis is influenced by various factors, including coelomogenesis, genetic predispositions, immune system disorders, environmental factors, and lifestyle changes [10]. Indeed, peritoneal (coelomic) cells or other non-endometrial cells transform into endometrial-like cells, possibly under hormonal or environmental influence [11]. Having a close relative with endometriosis significantly increases the risk, indicating a strong hereditary component. Certain gene variants have been associated with higher susceptibility [11]. Immune dysfunction may impair the body’s ability to clear endometrial tissue that migrates outside the uterus, allowing ectopic endometrial implants to persist [11]. Estrogen dependence, endocrine disruptors, obesity, and exposure to environmental toxins are associated with increased risk and may aggravate symptom severity [12, 13]. Furthermore, early menarche, frequent or heavy menstrual cycles, and low body mass index can heighten risk. Reproductive history, such as never giving birth and delayed menopause, also plays a role [14, 15]. The primary treatment modalities for endometriosis are pharmacotherapy and surgery, with laparoscopy being the main surgical approach [16, 17]. However, current non-surgical treatments, such as non-steroidal anti-inflammatory drugs, oral contraceptive pills, and hormonal therapies, often have limited efficacy. Approximately 25%−50% of patients discontinue these treatments due to adverse medication effects (e.g., spotting, nausea, breast tenderness, weight gain, headache, mood changes, hot flashes, and skin issues) [18, 19]. Given these challenges with conventional treatments, self-care becomes a relevant alternative in endometriosis management due to the significant limitations of current pharmacological and surgical treatments, including incomplete symptom relief, adverse effects, recurrence, and limited long-term solutions [4, 20]. Still, endometriosis-related literacy is necessary for optimal self-care, but it is necessary to evaluate that literacy to determine the gaps and barriers to optimal performance.

A knowledge, attitude, and practices (KAP) survey is a useful survey tool that offers a structured method for evaluating and understanding the awareness, perceptions, and behaviors of individuals regarding specific health issues [21–23]. In the case of endometriosis, a significant gap exists in current research, which largely focuses on immediate treatment and clinical aspects of the condition, often overlooking long-term management, i.e., patients’ sustained symptom monitoring, treatment adherence, and lifestyle or self-care adjustments beyond initial medical interventions [24, 25].

To date, most KAP studies on endometriosis have focused on healthcare professionals, resulting in a notable lack of understanding from the patients’ perspective [26, 27]. Patient-focused KAP research could help identify the knowledge gaps, misconceptions, and misunderstandings that could constitute barriers to the optimal performance of endometriosis self-care. This research aims to fill this void by investigating the knowledge, attitudes, and practices of patients themselves regarding endometriosis and its long-term management.

## Methods

### Study design and participants

This cross-sectional study was conducted at Fujian Maternity and Child Health Hospital between August and December 2023. Patients with a confirmed diagnosis of endometriosis were identified through the electronic medical record (EMR) system, and those who agreed to participate were included using a convenience sampling method. All eligible patients diagnosed with endometriosis during the study period at the hospital were invited to participate in the survey. The inclusion criterion was patients who conformed to the diagnostic standards outlined in the “Guidelines for the Diagnosis and Treatment of Endometriosis” by the Chinese Medical Association. The exclusion criteria were (1) patients with intellectual or psychiatric impairments, (2) patients unable to comply with the study protocols, or (3) patients who could not be contacted. The study was approved by the Ethics Committee of Fujian Maternity and Child Health Hospital [IRB NO.(2024KY094)]. All participants provided written informed consent.

### Questionnaire

The questionnaire for this study was designed with inspiration from authoritative sources, including the “2018 French Association of Obstetricians and Gynecologists/National Health Management Guidelines for Endometriosis” and the “Expert Consensus on Long-Term Management of Endometriosis in China” as published in the “Chinese Journal of Obstetrics and Gynecology” in 2018 [28]. After an initial draft, the questionnaire underwent content validation by two specialists in obstetrics and gynecology, each with over 20 years of clinical experience and holding senior professional titles. Their feedback was used to refine the items, ensuring relevance and clarity [22]. The internal consistency of the questionnaire was acceptable, as shown by Cronbach’s α = 0.767 (Cronbach’s α >0.7 is considered acceptable [29]).

The final version of the questionnaire, provided in Chinese (a translated English version is attached as an Appendix), covered five key dimensions and consisted of a total of 44 items. These included 12 items for basic information, 13 items for assessing knowledge, 8 items for attitudes, and 7 items for practices. The responses were scored based on selected options. For the knowledge section, responses ranged from “very familiar” (2 points) to “uncertain” (0 points). The total knowledge score ranged from 0 to 24. The attitude section utilized a 5-point Likert scale. Items A3, A4, A5, A6, A7, and A8 were scored from “very positive” (5 points) to “very negative” (1 point). Items A1 and A2 were reverse scored from “very positive” (1 point) to “very negative” (5 points). The practice dimension followed a uniform scoring pattern, with each item scored from 5 (very positive) to 1 (very negative), resulting in a total score range of 7 to 35. The KAP dimension scores were converted to percentages. They were categorized according to Bloom’s cut-offs (< 60%, 60–79%, and *≥* 80%) [30–32]. Hence, knowledge was categorized as inadequate, moderate, or sufficient, attitude as negative, neutral, or positive, and practice as negative, moderate, or positive. Data collection was conducted by administering questionnaires to eligible participants in person at hospital clinics and remotely through telephone interviews.

### Questionnaire quality control

Three researchers were enlisted and underwent comprehensive training on the protocol, interview techniques, and data collection to standardize the survey process and to safeguard the quality and reliability of the survey. Post-training, these researchers engaged with potential participants, elucidating the study’s objectives and its importance. Patients who expressed their consent to partake in the survey were then interviewed via telephone, during which the researchers administered the questionnaire. This approach ensured that the data collection was both consistent and reliable.

#### Statistical analysis

Data analysis was conducted using SPSS 22.0 (IBM, Armonk, NY, USA). Continuous data are presented as means and standard deviations (SD), while categorical data are expressed as n (%). Continuous variables underwent a normality test, using the t-test for normally distributed data and the Wilcoxon-Mann-Whitney test for non-normally distributed data when comparing two groups. For three or more groups with normally distributed continuous variables and uniform variance, ANOVA was used for comparisons, while the Kruskal-Wallis test was employed for non-normally distributed data. A Spearman correlation analysis was performed to assess potential associations among key sociodemographic variables (such as education, income, and ethnicity). Structural equation modeling (SEM) was employed to investigate the relationships among knowledge, attitude, and practice. The initial selection of variables in the SEM was guided by both the conceptual KAP framework and the results of univariate analyses. The hypotheses of the KAP framework included that knowledge directly impacts attitude and practice, while attitude directly impacts practice. The demographic characteristics were selected according to univariate analysis. Specifically, characteristics that showed significant differences in KAP scores (e.g., education and knowledge) were included in the model. The assignments of demographic characteristics in the SEM analysis are described in Table S1. Ordered characteristics were coded in ascending order. The categorical variables were assigned with 1 for Yes and 0 for No. Specifically, “Prefer not to disclose” for income was considered as a missing value and assigned as 3 based on the mean substitution method [33]. The SEM was further refined by removing nonsignificant paths identified in the initial model to improve overall model fit. A two-sided P-value less than 0.05 was considered statistically significant. Model assumptions were assessed by examining standard goodness-of-fit indices, including CMIN/DF, RMSEA, SRMR, TLI, CFI, and IFI. As the model included categorical variables that do not meet the multivariate normality assumption of the Maximum Likelihood (ML) estimator in AMOS, the bootstrap method was used to estimate standard errors and confidence intervals more robustly.

## Results

### Characteristics of the participants

Initially, a total of 301 patients participated in this study, and none of them were excluded after enrollment. Their mean age was 32.26 ± 6.75 years, 203 (67.44%) had college/bachelor’s degree, 217 (72.09%) were employed, 220 (73.09%) were married, 137 (45.51%) had children, 180 (59.8%) were diagnosed with endometriosis for less than 1 year, and 63 (20.93%) had a female relative with endometriosis.

### KAP dimensions

The mean knowledge, attitude, and practice scores were 13.28 ± 5.98 (possible range: 0–24), 28.23 ± 2.38 (possible range: 8–40), and 22.56 ± 4.76 (possible range: 7–35), respectively. Significantly, the participants with different education levels (*P* < 0.001), ethnicity (*P* = 0.015), having children (*P* = 0.025), duration of endometriosis diagnosis (*P* < 0.001), and diagnosed relatives (*P* < 0.001) were more likely to have different knowledge scores. Effect sizes (Cohen’s d or η², where applicable) are presented in Table 1 to provide additional context for interpreting between-group differences. Patients with different average monthly per capita incomes (*P* < 0.001), drinking habits (*P* < 0.001), health insurance (*P* < 0.001), and diagnosed relatives (*P* < 0.001) were more likely to have different attitude scores. Those who differ by education levels (*P* < 0.001), ethnicity (*P* = 0.004), employment status (*P* < 0.001), average monthly per capita income (*P* < 0.001), marital status (*P* = 0.017), drinking habit (*P* = 0.001), health insurance (*P* = 0.005), duration of endometriosis diagnosis (*P* = 0.002), and diagnosed relatives (*P* < 0.001) were more likely to have different practice scores (Table 1).

**Table 1:** Demographic characteristics and KAP scores

*N* = 301	*N* (%)	Knowledge score		Attitude score		Practice score	
		Mean ± SD	*P* (effect size)	Mean ± SD	*P* (effect size)	Mean ± SD	*P* (effect size)
Total score		13.28 ± 5.98		28.23 ± 2.38		22.56 ± 4.76	
Age (years)	32.26 ± 6.75						
Education			< 0.001 (24.628)		0.438 (2.908)		< 0.001 (32.434)
Junior high school and below	21(6.98)	8.80 ± 4.93		28.09 ± 1.72		17.57 ± 4.74	
High school/technical school	68(22.59)	12.25 ± 5.85		28.05 ± 2.57		22.35 ± 4.30	
College/bachelor’s degree	203(67.44)	13.82 ± 5.78		28.32 ± 2.36		22.94 ± 4.59	
Graduate and above	9(2.99)	19.33 ± 6.30		27.55 ± 2.83		27.22 ± 3.34	
Ethnicity			0.015 (272.000)		0.977 (734.500)		0.004 (181.500)
Han	296(98.34)	13.39 ± 5.93		28.22 ± 2.40		22.71 ± 4.58	
Minority ethnic group	5(1.66)	6.8 ± 5.35		28.2 ± 1.09		13.2 ± 6.22	
Employment			0.221 (6.562)		0.755 (0.739)		< 0.001 (24.912)
Employed	217(72.09)	13.70 ± 6.14		28.18 ± 2.46		23.14 ± 4.49	
Unemployed	27(8.97)	11.77 ± 5.69		28.51 ± 1.45		18.96 ± 4.81	
Other	57(18.94)	12.40 ± 5.33		28.24 ± 2.45		22.05 ± 4.98	
Monthly Household Income, Yuan			0.090 (8.034)		< 0.001 (43.911)		< 0.001 (29.196)
< 2000	/	/	/	/	/	/	/
2000–5000	47(15.61)	12.48 ± 6.04		28.65 ± 2.11		20.74 ± 4.59	
5000–10,000	95(31.56)	13.34 ± 6.11		29.31 ± 1.90		21.52 ± 5.26	
10,000–20,000	13(4.32)	13.15 ± 4.25		28.38 ± 3.47		26.92 ± 2.75	
> 20,000	11(3.65)	9.27 ± 2.37		26.81 ± 1.88		24.45 ± 0.52	
Prefer not to disclose	135(44.85)	13.85 ± 6.11		27.40 ± 2.35		23.34 ± 4.32	
Marital status			0.089 (3.199)		0.281 (1.254)		0.017 (6.214)
Divorced/unmarried	81(26.91)	14.02 ± 5.22		28.43 ± 2.15		23.70 ± 4.23	
Married	220(73.09)	13.00 ± 6.22		28.15 ± 2.46		22.14 ± 4.88	
Have children			0.025 (12912.000)		0.515 (11720.00)		0.938 (11176.00)
Yes	137(45.51)	12.51 ± 5.70		28.16 ± 2.59		22.53 ± 4.62	
No	164(54.49)	13.92 ± 6.14		28.27 ± 2.20		22.58 ± 4.88	
Smoking			0.124 (2.441)		0.546 (5.982)		0.050 (4.502)
No	296(98.34)	13.21 ± 5.97		28.21 ± 2.38		22.49 ± 4.75	
Yes	5(1.66)	17.4 ± 5.81		28.8 ± 2.77		26.6 ± 3.28	
Drinking			0.248 (2.786)		< 0.001 (41.293)		0.001 (15.232)
Never drink	203(67.44)	13.74 ± 6.10		27.64 ± 2.27		23.32 ± 4.52	
Used to drink	63(20.93)	12.66 ± 5.08		29.15 ± 1.97		21.25 ± 4.75	
Still drink now	35(11.63)	11.68 ± 6.54		29.94 ± 2.33		20.48 ± 5.07	
Health insurance			0.501 (1665.500)		< 0.001 (726.500)		0.005 (2725.500)
Yes	288(95.68)	13.35 ± 6.08		28.33 ± 2.33		22.42 ± 4.72	
No	13(4.32)	11.69 ± 2.32		25.76 ± 2.27		25.69 ± 4.69	
Duration of endometriosis diagnosis			< 0.001 (28.042)		0.084 (6.656)		0.002 (15.401)
< 1 year	180(59.8)	12 ± 5.95		28.17 ± 2.07		21.68 ± 5.18	
1 ~ 3 years	74(24.58)	14.02 ± 5.21		27.94 ± 3.01		24.12 ± 3.58	
3 ~ 5 years	17(5.65)	15.70 ± 4.22		29.35 ± 1.76		22.58 ± 3.64	
> 5 years	30(9.97)	17.76 ± 6.11		28.6 ± 2.54		23.96 ± 3.95	
Have female relatives with endometriosis			< 0.001 (37.365)		< 0.001 (30.533)		< 0.001 (49.163)
Yes	63(20.93)	15.44 ± 5.23		29.33 ± 2.43		23.23 ± 4.00	
No	153(50.83)	14.18 ± 5.91		27.57 ± 2.29		23.94 ± 4.28	
Not sure	85(28.24)	10.05 ± 5.36		28.57 ± 2.12		19.56 ± 4.78	

*Abbreviations*: *KAP* Knowledge, attitude, and practice, *SD* Standard deviation

The distribution of the responses to the knowledge items showed that the two questions with the highest proportion choosing the “not clear” option were “*Laparoscopic examination is currently recognized as the optimal diagnostic method for endometriosis.*” (K6) with 40.86% and “*Currently*,* the preferred treatment involves laparoscopic surgery combined with drug therapy.*” (K7) with 36.12% (Table S2). The responses to the attitude items revealed notable perceptions toward endometriosis. Many participants reported feelings of worry, fear, anxiety, and unease, although a considerable proportion remained neutral on these points. In contrast, a clear majority believed that regular check-ups and active exercise could help reduce the risk of developing endometriosis. Views on the adequacy of hospital education and long-term management were more divided, with many respondents expressing neutrality. Importantly, most participants emphasized that understanding and encouragement from family members played a vital role in supporting patients (Table S3). Patient practices showed substantial variation. Many participants regularly adhered to follow-up appointments and maintained their prescribed medication schedules. However, engagement in educational activities related to endometriosis and its long-term management was considerably lower. While some individuals occasionally sought information, a notable portion rarely or never participated in such activities, highlighting a gap between clinical adherence and active self-education (Table S4).

### Correlations

To explore the correlation between knowledge, attitude, and practice, a correlation analysis was conducted, and the results showed that knowledge has a positive correlation with attitude (*r* = 0.2439, *P* < 0.001) as well as a positive correlation with practice (*r* = 0.3918, *P* < 0.001). However, the correlation between attitude and practice was not significant (*r* = −0.0505, *P* = 0.3829) (Table 2). As part of the model validation, we also conducted a Spearman correlation analysis to assess potential intercorrelations among key sociodemographic variables, including education, income, and ethnicity. The results showed all coefficients were below 0.3, indicating weak correlations and supporting their independent inclusion.

**Table 2:** Correlation analysis

	Knowledge	Attitude	Practice
Knowledge	1		
Attitude	0.2439 (*P*<0.001)	1	
Practice	0.3918 (*P*<0.001)	−0.0505 (*P* = 0.3829)	1

*Abbreviations*: *K* Knowledge, *A* Attitude, *P* Practice

### SEM analysis

**Fig. 1:**
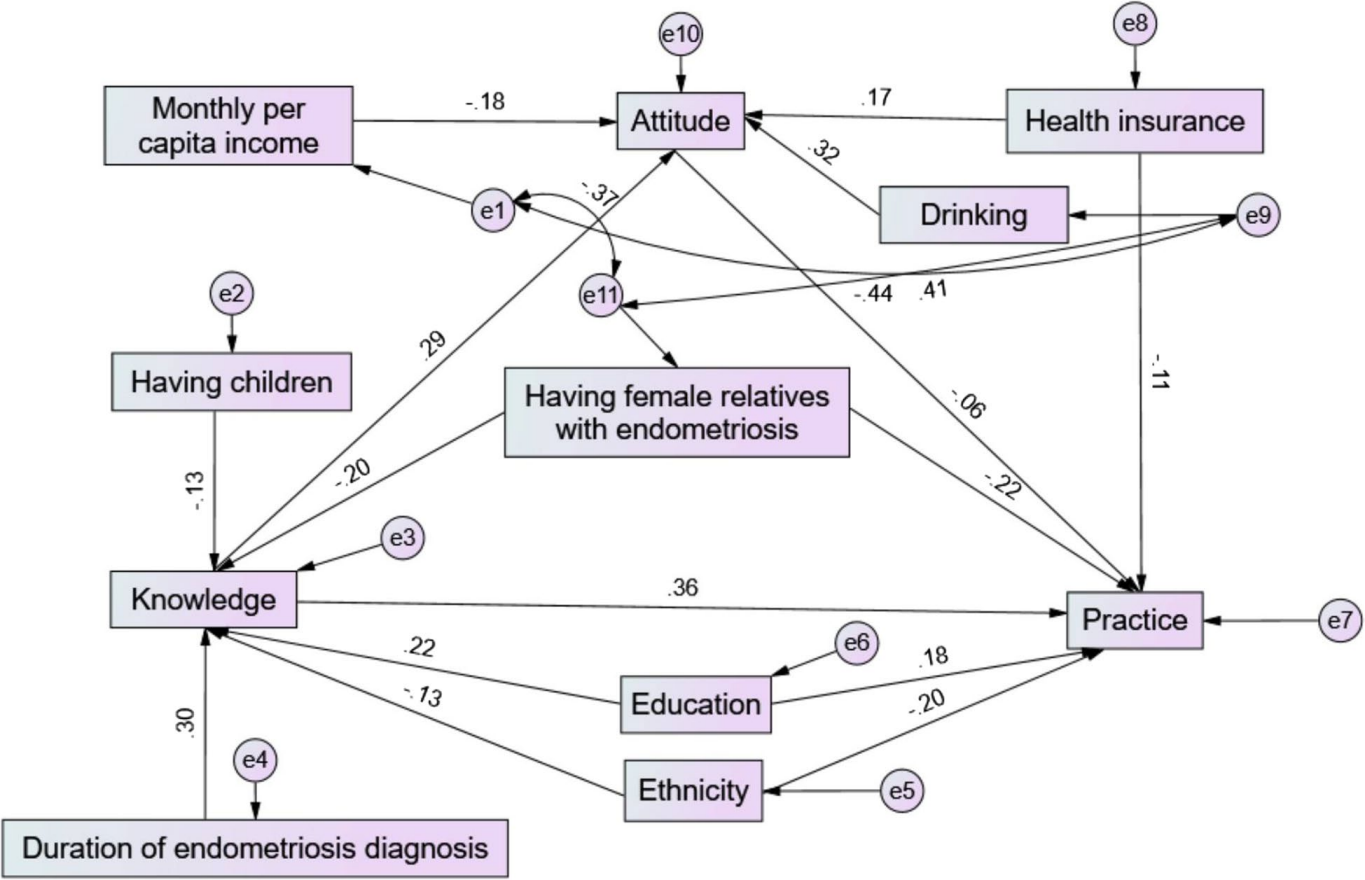
SEM model

The fit of the final SEM model was acceptable (CMIN/DF = 2.306, RMSEA = 0.066, SRMR = 0.068, TLI = 0.852, CFI = 0.903, and IFI = 0.907) (Table 3 and Table S5). Knowledge directly influenced attitude, and knowledge directly influenced practice. Duration of endometriosis and education had significant positive direct effects on knowledge, while having children, ethnicity, and having female relatives with endometriosis had negative direct effects. Drinking and health insurance positively influenced attitude, while having children, ethnicity, having female relatives with endometriosis, and income negatively influenced attitude. Duration of endometriosis diagnosis and education positively influenced practice, while having children, ethnicity, having female relatives with endometriosis, and health insurance negatively influenced practice. The path from attitude to practice remained nonsignificant, suggesting that attitude did not directly translate into reported behaviors (Table 4 and Fig. 1).

**Table 3:** SEM model fit

Indicators	Reference	Results
CMIN/DF	< 3 Good	2.306
RMSEA	< 0.08 Good	0.066
SRMR	< 0.08 Good	0.068
TLI	> 0.8 Good	0.852
CFI	> 0.8 Good	0.903
IFI	> 0.8 Good	0.907

*Abbreviations*: *RMSEA* Root mean square error of approximation, *SRMR* Standardized root mean square residual, *TLI* Tucker-Lewis index, *CFI* Comparative fit index

**Table 4:** Results of SEM analysis

Model paths	Standardized Total effects		Standardized direct effects		Standardized indirect effects	
	β (95%CI)	*P*	β (95%CI)	*P*	β (95%CI)	*P*
Knowledge→Attitude	0.287 (0.197,0.391)	0.014	0.287 (0.197,0.391)	0.014		
Attitude→Practice	−0.064 (−0.179,0.047)	0.247	−0.064 (−0.179,0.047)	0.247		
Knowledge→Practice	0.342 (0.237,0.452)	0.005	0.361 (0.254,0.473)	0.005	−0.019 (−0.060,0.014)	0.227
Duration of endometriosis diagnosis→Knowledge	0.300 (0.195,0.420)	0.006	0.300 (0.195,0.420)	0.006		
Having children→Knowledge	−0.127 (−0.268,−0.035)	0.010	−0.127 (−0.268,−0.035)	0.010		
Ethnicity→Knowledge	−0.127 (−0.232,−0.022)	0.010	−0.127 (−0.232,−0.022)	0.010		
Education→Knowledge	0.217 (0.115,0.310)	0.009	0.217 (0.115,0.310)	0.009		
Having female relatives with endometriosis→Knowledge	−0.202 (−0.305,−0.115)	0.004	−0.202 (−0.305,−0.115)	0.004		
Duration of endometriosis diagnosis→Attitude	0.086 (0.056,0.149)	0.003			0.086 (0.056,0.149)	0.003
Having children→Attitude	−0.037 (−0.085,−0.006)	0.015			−0.037 (−0.085,−0.006)	0.015
Ethnicity→Attitude	−0.037 (−0.078,−0.008)	0.008			−0.037 (−0.078,−0.008)	0.008
Education→Attitude	0.062 (0.031,0.102)	0.008			0.062 (0.031,0.102)	0.008
Having female relatives with endometriosis→Attitude	−0.058 (−0.100,−0.031)	0.004			−0.058 (−0.100,−0.031)	0.004
Monthly per capita income→Attitude	−0.176 (−0.292,−0.052)	0.009	−0.176 (−0.292,−0.052)	0.009		
Drinking→Attitude	0.318 (0.215,0.440)	0.008	0.318 (0.215,0.440)	0.008		
Health insurance→Attitude	0.169 (0.043,0.268)	0.016	0.169 (0.043,0.268)	0.016		
Duration of endometriosis diagnosis→Practice	0.103 (0.056,0.159)	0.006			0.103 (0.056,0.159)	0.006
Having children→Practice	−0.044 (−0.092,−0.012)	0.010			−0.044 (−0.092,−0.012)	0.010
Ethnicity→Practice	−0.242 (−0.377,−0.074)	0.015	−0.198 (−0.314,−0.074)	0.012	−0.044 (−0.080,−0.010)	0.008
Education→Practice	0.258 (0.143,0.362)	0.013	0.183 (0.082,0.285)	0.010	0.074 (0.037,0.109)	0.009
Having female relatives with endometriosis→Practice	−0.293 (−0.426,−0.192)	0.007	−0.224 (−0.331,−0.117)	0.014	−0.067 (−0.118,−0.038)	0.003
Monthly per capita income→Practice	0.011 (−0.005,0.058)	0.119			0.011 (−0.005,0.058)	0.119
Drinking→Practice	−0.021 (−0.056,0.012)	0.218			−0.021 (−0.056,0.012)	0.218
Health insurance→Practice	−0.123 (−0.251,−0.017)	0.030	−0.112 (−0.255,−0.007)	0.041	−0.011 (−0.033,0.005)	0.198

*Abbreviations*: *CI* Confidence interval

## Discussion

This study showed that patients with endometriosis had inadequate knowledge, moderate attitudes, and practices towards endometriosis and its long-term management. The SEM analysis showed that knowledge and attitudes influenced practices regarding endometriosis and its long-term management. The patients’ point of view is pivotal in developing effective and personalized long-term management strategies for endometriosis.

The findings of our study shed light on patients’ knowledge, attitudes, and practices regarding endometriosis and its long-term management. Such gaps may stem from delays in seeking medical attention for endometriosis diagnosis, insufficient symptom assessment by general practitioners, and obstacles to effective communication between patients and healthcare professionals [34].

Correlation analysis revealed positive associations between knowledge, attitude, and practice in endometriosis management. However, SEM indicated that attitude did not significantly mediate the relationship between knowledge and practice. Instead, knowledge directly influenced both attitude and practice, while the attitude–practice pathway was not significant. This suggests that improved knowledge can translate into better practices without necessarily altering attitudes, likely reflecting pragmatic health concerns or external influences [35]. Key predictors of knowledge and practice included education, disease duration, and family history, while having children and minority ethnicity were negatively associated. Higher educational attainment was strongly linked to greater knowledge and better practices, consistent with prior research. Minority groups reported lower knowledge and practice scores, possibly due to language barriers, limited health education access, or cultural beliefs regarding reproductive health. Family history of endometriosis appeared to lower awareness and engagement, likely because symptoms were normalized within families [36–38]. Income also shaped attitudes and practices, highlighting socioeconomic disparities in health literacy and access to gynecological care [38–40]. Together, these results reinforce knowledge as the central driver of attitudes and behaviors, while pointing to structural and cultural factors that shape patient outcomes [41]. Targeted strategies are therefore recommended, including education tailored to different literacy levels, culturally sensitive interventions, economic support for disadvantaged groups, and family-focused awareness initiatives. Health interventions frequently use plain language, visual materials, and in-person teaching for individuals with low literacy. Programs may deliver bilingual educational brochures, video-based lessons, or face-to-face sessions, matching both language proficiency and literacy requirements. Focus group discussions and needs assessments often help customize content to participants’ literacy and language backgrounds [42]. Addressing these factors may help improve disease understanding, promote positive health practices, and reduce disparities in endometriosis management [4, 43]. These findings align with previous studies [44] emphasizing the importance of raising awareness about endometriosis symptoms to facilitate early diagnosis and management. Recommendations include targeted public health campaigns, particularly focusing on symptom recognition and the importance of seeking timely medical consultation [45].

Regarding attitudes towards endometriosis, our study identified areas where participants displayed varying levels of agreement. Notably, participants expressed concern and anxiety about the disease, highlighting its emotional impact and suggesting the necessity of providing psychological support and counseling services to patients. On a positive note, a significant proportion of patients felt mentally prepared for the long-term struggle against the disease, indicating resilience among them. However, a substantial number remained neutral regarding their unease about the complexity of endometriosis, emphasizing the need for educational interventions to address these uncertainties. Additionally, a minority believed that having sufficient knowledge about the disease is helpful for long-term management, indicating a potential gap in understanding the value of knowledge. Recommendations include integrating psychological support services and targeted educational programs to address attitude-related concerns [17, 46].

In the practice dimension, participants’ behaviors related to the management of endometriosis were assessed. Notably, a significant portion reported actively learning about endometriosis knowledge and paying attention to their diet. These practices align with recommendations for patient self-education and lifestyle modifications to manage endometriosis symptoms. However, a substantial number of participants sometimes made efforts to adjust their emotions, indicating potential room for improvement in addressing the emotional aspects of the disease. Additionally, while a considerable proportion followed medical advice for medication treatment, there is room for improvement in adherence to medical advice regarding pharmacotherapy and lifestyle changes. To improve patient practices, healthcare providers should emphasize the importance of emotional well-being, adherence to treatment plans, and the adoption of healthy lifestyle practices. Furthermore, organized educational activities by medical institutions can further encourage patient engagement and knowledge acquisition [47–49]. Based on the findings, we suggest practical recommendations such as tailoring education programs to diverse educational and cultural backgrounds, incorporating family-oriented health education, and strengthening psychological support and follow-up services to improve long-term disease management.

The limitations of this study should be acknowledged. First, it was conducted at a single healthcare institution, which may limit generalizability. Second, convenience sampling from EMR records and voluntary participation may have introduced selection and self-selection bias, limiting representativeness. Third, reliance on self-reported data carries risks of recall and social desirability bias, potentially inflating health-promoting behaviors or underreporting negative attitudes, thus affecting KAP scores. Fourth, treating “Prefer not to disclose” income responses as missing and imputing them by mean substitution may have biased variable relationships and underestimated errors [33]. Future research with more diverse samples and objective measures would strengthen the robustness of findings on patients’ knowledge, attitudes, and practices regarding endometriosis management.

## Conclusion

In conclusion, this study demonstrated inadequate knowledge, neutral attitudes, and moderate practices regarding endometriosis and its long-term management. To improve patient outcomes, healthcare providers must prioritize education and awareness campaigns on endometriosis, especially targeting individuals with lower education levels, diverse ethnic backgrounds, and limited resources, to enhance their understanding and engagement in effective management practices. The results could also help inform policy, guiding future research or shaping clinical guidelines for the long-term management of endometriosis.

## Supplementary Information

Supplementary Material 1.

Supplementary Material 2.

Supplementary Material 3.

Supplementary Material 4.

Supplementary Material 5.

Supplementary Material 6.

## Data Availability

All data generated or analyzed during this study are included in this article and supplementary information files.

## Abbreviations

KAP: Knowledge, Attitude, and Practices
SD: Standard deviations
SEM: Structural equation modeling
