# Supplementary material for: Patients’ knowledge, attitudes, and practices concerning endometriosis and its long-term management
Source: BMC Womens Health. 2025 Nov 28;25:633. doi: 10.1186/s12905-025-04187-z (PMC12750744; doi:10.1186/s12905-025-04187-z)
Supplement: Supplementary file 1 — Supplementary Material 1. [file 12905_2025_4187_MOESM1_ESM.docx]

| **Dear Participant,**  We are researchers from XXX and cordially invite you to participate in our research study. The aim of this study is to understand patients' awareness, attitudes, and practices regarding endometriosis and its long-term management. This will provide a basis for developing scientific intervention strategies that could potentially help more people in the future and improve their health conditions. Your participation in this study is entirely voluntary, and the study has been reviewed and approved by the ethics review committee. If you agree to participate, please refer to the instructions below.   1. Please complete the questionnaire. There are no right or wrong answers; simply provide responses based on your actual situation. If you have any questions during the process, feel free to ask us. After completion, please submit the questionnaire promptly. 2. This research involves a simple questionnaire survey and will not cause any harm to your physical or psychological health. However, it will involve some private matters such as your gender and age, which we will keep strictly confidential. Rest assured, your information will not be disclosed. 3. As a participant, you may access information related to the study and its progress at any time. If you decide to withdraw from the study, please inform us, and your data will not be included in the research results.   Finally, we sincerely thank you for taking the time out of your busy schedule to support our scientific research!  □ I am informed and agree to the use of the collected data for scientific research. Informed Consent Signature: Participation Date: Year Month Day |
| --- |

| **Section One: Basic Information** | |
| --- | --- |
| **1. Age： years old。** | |
| **2.Education：** | a. primary school or below  b. middle school  c. high school/ technical school  d. college/ bachelor’s degree  e. master’s degree and above |
| **3. Ethnicity** | a. han  b. minority |
| **4. Employment status：** | a. employed  b. unemployed  c. retired  d. self-employed  f. other |
| **5.** **Your average monthly per capita household income in the past year (including in-kind income and rental income, etc.): ______ Yuan** | a.<2000  b.2000-5000  c.5000-10000  d.10000-20000  e.>20000  f. prefer not to disclose |
| **6.Marital status：** | a. single  b. married  c. divorced  d. widowed |
| **7. Do you have children：** | a. yes  b. no |
| **8.** **Do you have a smoking habit?** | a. never smoked  b. former smoker  c. currently smoking |
| **9.** **Do you have a drinking habit?** | a. never drank  b. former drinker  c. currently drinking |
| **10.** **Do you have medical or other commercial insurance?** | a. yes  d. no |
| **11.** **How long have you been diagnosed with endometriosis?** | a.<1 year  b.1~3 years  c.3~5 years  d.>5 years |
| **12.** **Do you have female relatives with endometriosis?** | a. yes  d. no  c. not sure |

| **Section Two: Knowledge of Endometriosis and Its Long-Term Management** | | | |
| --- | --- | --- | --- |
| 1. Endometriosis (EMT) refers to the growth of endometrial tissue (glands and stroma) outside the uterine cavity and myometrium. | a. very familiar | b. heard of it | c. unfamiliar |
| 2. Endometriosis is mainly characterized by progressively worsening secondary dysmenorrhea, common among women of childbearing age, 25-45 years old. | a. very familiar | b. heard of it | c. unfamiliar |
| 3. Some endometriosis patients exhibit no symptoms. | a. very familiar | b. heard of it | c. unfamiliar |
| 4. Endometriosis is a chronic disease with an infertility rate as high as 40%. | a. very familiar | b. heard of it | c. unfamiliar |
| 5. If symptoms like progressively worsening dysmenorrhea, abnormal menstruation, severe abdominal pain, painful intercourse, or difficulty conceiving appear, it is necessary to seek medical attention promptly. | a. very familiar | b. heard of it | c. unfamiliar |
| 6. Laparoscopy is currently recognized as the best method for diagnosing endometriosis. | a. very familiar | b. heard of it | c. unfamiliar |
| 7. The best treatment approach currently is a combination of laparoscopic surgery and medication. | a. very familiar | b. heard of it | c. unfamiliar |
| 8. Apart from radical surgery, the recurrence rate of endometriosis is relatively high. | a. very familiar | b. heard of it | c. unfamiliar |
| 9. Patients with endometriosis should pay attention to their symptoms and disease progression in daily life, and those on long-term medication should observe for any adverse drug reactions. | a. very familiar | b. heard of it | c. unfamiliar |
| 10. The preventive effects for endometriosis are limited, but timely treatment of genital tract diseases, oral contraceptives, and regular exercise may reduce the risk of developing the disease. | a. very familiar | b. heard of it | c. unfamiliar |
| 11. To relieve pain, patients should rest in bed as much as possible during menstruation; severe cases may require oral or rectal anti-inflammatory painkillers. | a. very familiar | b. heard of it | c. unfamiliar |
| 12. Patients should maintain regular physical exercise and a proper diet in their daily lives. | a. very familiar | b. heard of it | c. unfamiliar |
| 13. Patients should follow up according to medical advice, usually once every 3-6 months. | a. very familiar | b. heard of it | c. unfamiliar |

| **Section Three: Attitudes Towards Endometriosis and Its Long-Term Management** | | | | | |
| --- | --- | --- | --- | --- | --- |
| 1. Feel worried, fearful, and anxious about endometriosis. | a. strongly agree | b. agree | c. neutral | d. disagree | e. strongly disagree |
| 2. Feel uneasy about the complexity of endometriosis and its impact on life. | a. strongly agree | b. agree | c. neutral | d. disagree | e. strongly disagree |
| 3. Have prepared mentally for a long-term struggle with the disease. | a. strongly agree | b. agree | c. neutral | d. disagree | e. strongly disagree |
| 4. Believe that having adequate knowledge about the disease helps in the long-term management of endometriosis. | a. strongly agree | b. agree | c. neutral | d. disagree | e. strongly disagree |
| 5. Believe that with active treatment and daily management, the disease can be overcome. | a. strongly agree | b. agree | c. neutral | d. disagree | e. strongly disagree |
| 6. Believe that although the disease is difficult to prevent, it is necessary to reduce the risk of onset through regular check-ups and active exercise. | a. strongly agree | b. agree | c. neutral | d. disagree | e. strongly disagree |
| 7. Believe that hospitals do not provide enough education on endometriosis and its long-term management. | a. strongly agree | b. agree | c. neutral | d. disagree | e. strongly disagree |
| 8. Believe that the understanding and encouragement from family members are crucial for building confidence in overcoming the disease and avoiding anxiety and unease. | a. strongly agree | b. agree | c. neutral | d. disagree | e. strongly disagree |

| **Section Four: Practices Regarding Endometriosis and Its Long-Term Management** | | | | | |
| --- | --- | --- | --- | --- | --- |
| 1. Actively learn about endometriosis and its long-term management. | a. always | b. often | c. occasionally | d. rarely | e. never |
| 2. Pay attention to diet, eating more fresh vegetables and fruits, salmon, and walnuts, while consuming less high-fat meat products. | a. always | b. often | c. occasionally | d. rarely | e. never |
| 3. Make efforts to adjust mood and actively cope with the physical and emotional challenges that endometriosis may bring. | a. always | b. often | c. occasionally | d. rarely | e. never |
| 4. Regularly undergo follow-up check-ups. | a. always | b. often | c. occasionally | d. rarely | e. never |
| 5. Regularly engage in physical exercise. | a. always | b. often | c. occasionally | d. rarely | e. never |
| 6. Actively participate in educational activities on endometriosis and its long-term management organized by any medical institution. | a. always | b. often | c. occasionally | d. rarely | e. never |
| 7. Follow medical advice for medication treatment. | a. always | b. often | c. occasionally | d. rarely | e. never |
