# Supplementary material for: Patients’ knowledge, attitudes, and practices concerning endometriosis and its long-term management
Source: BMC Womens Health. 2025 Nov 28;25:633. doi: 10.1186/s12905-025-04187-z (PMC12750744; doi:10.1186/s12905-025-04187-z)
Supplement: Supplementary file 2 — Supplementary Material 2. [file 12905_2025_4187_MOESM2_ESM.docx]

Table S1. Assignments of demographic characteristics of SEM

| Characteristics | Assignments |
| --- | --- |
| **Education** |  |
| Junior high school and below | 1 |
| High school/ technical school | 2 |
| College/ bachelor's degree | 3 |
| Graduate and above | 4 |
| **Ethnicity** |  |
| Han | 0 |
| Minority ethnic group | 1 |
| **Monthly Household Income, Yuan** |  |
| <2000 | 1 |
| 2000-5000 | 2 |
| 5000-10000 | 3 |
| 10000-20000 | 4 |
| >20000 | 5 |
| Prefer not to disclose | 3 |
| **Marital status** |  |
| Divorced /unmarried | 0 |
| Married | 1 |
| **Have children** |  |
| Yes | 1 |
| No | 0 |
| **Drinking** |  |
| Never drink | 0 |
| Used to drink | 1 |
| Still drink now | 2 |
| **Health insurance** |  |
| Yes | 1 |
| No | 0 |
| **Duration of endometriosis diagnosis** |  |
| <1 year | 1 |
| 1~3 years | 2 |
| 3~5 years | 3 |
| >5 years | 4 |
| **Have female relatives with endometriosis** |  |
| Yes | 1 |
| No | 0 |
| Not sure | 0 |
