# Supplementary material for: Patients’ knowledge, attitudes, and practices concerning endometriosis and its long-term management
Source: BMC Womens Health. 2025 Nov 28;25:633. doi: 10.1186/s12905-025-04187-z (PMC12750744; doi:10.1186/s12905-025-04187-z)
Supplement: Supplementary file 3 — Supplementary Material 3. [file 12905_2025_4187_MOESM3_ESM.docx]

**Table S2. Distribution of knowledge dimension responses**

| **Knowledge** | **N(%)** | | |
| --- | --- | --- | --- |
|  | **a. very familiar** | **b. heard of** | **c. not clear** |
| **1.** **Endometriosis refers to the growth of endometrial tissue (glands and stroma) outside the uterine cavity and uterine muscle layer.** | 45(14.95) | 196(65.12) | 60(19.93) |
| **2.** **The main presentation of endometriosis is progressively worsening secondary dysmenorrhea, commonly observed in women of childbearing age, typically between 25 and 45 years old.** | 68(22.59) | 187(62.13) | 46(15.28) |
| **3.** **Some endometriosis patients may exhibit no symptoms.** | 51(16.94) | 151(50.17) | 99(32.89) |
| **4.** **Endometriosis is considered a chronic condition, with infertility rates reaching up to 40%.** | 65(21.59) | 150(49.83) | 86(28.57) |
| **5.** **If symptoms such as worsening menstrual pain, abnormal menstruation, severe abdominal pain, pain during intercourse, and difficulty getting pregnant occur, early medical consultation is advised.** | 103(34.22) | 159(52.82) | 39(12.96) |
| **6.** **Laparoscopic examination is currently recognized as the optimal diagnostic method for endometriosis.** | 65(21.59) | 113(37.54) | 123(40.86) |
| **7.** **Currently, the preferred treatment involves laparoscopic surgery combined with drug therapy.** | 68(22.59) | 124(41.2) | 109(36.21) |
| **8.** **Apart from radical surgery, endometriosis has a relatively high recurrence rate.** | 96(31.89) | 115(38.21) | 90(29.9) |
| **9.** **Endometriosis patients should pay attention to their symptoms in daily life, monitor disease progression, and individuals on long-term medication should observe any potential adverse drug reactions.** | 81(26.91) | 169(56.15) | 51(16.94) |
| **10.** **While preventive measures for endometriosis are limited, timely treatment of reproductive system abnormalities, oral contraceptives, and regular exercise may reduce the risk of occurrence.** | 47(15.61) | 189(62.79) | 65(21.59) |
| **11.** **To alleviate pain, patients should consider bed rest during menstruation, and those with severe symptoms may need to take anti-inflammatory and analgesic drugs orally or through rectal suppositories.** | 80(26.58) | 186(61.79) | 35(11.63) |
| **12.** P**atients should engage in regular physical exercise and maintain a balanced diet in their daily lives.** | 77(25.58) | 194(64.45) | 30(9.97) |
| **13.** **Patients are advised to follow medical recommendations, typically with follow-up appointments every 3 to 6 months.** | 116(38.54) | 141(46.84) | 44(14.62) |
