# Supplementary material for: Patients’ knowledge, attitudes, and practices concerning endometriosis and its long-term management
Source: BMC Womens Health. 2025 Nov 28;25:633. doi: 10.1186/s12905-025-04187-z (PMC12750744; doi:10.1186/s12905-025-04187-z)
Supplement: Supplementary file 4 — Supplementary Material 4. [file 12905_2025_4187_MOESM4_ESM.docx]

**Table S3. Distribution of attitude dimension responses**

| **Attitude** | **Strongly agree** | **Agree** | **Neutral** | **Disagree** | **Strongly disagree** |
| --- | --- | --- | --- | --- | --- |
| **1.** **Concerned, fearful, and anxious about endometriosis. (N)** | 14(4.65) | 143(47.51) | 101(33.55) | 43(14.29) | / |
| **2.** **Uneasy about the complexity of endometriosis and its impact on life. (N)** | 9(2.99) | 126(41.86) | 115(38.21) | 51(16.94) | / |
| **3.** **Mentally prepared for the long-term struggle against the disease. (P)** | 24(7.97) | 162(53.82) | 97(32.23) | 18(5.98) | / |
| **4.** **Believes that having sufficient knowledge about the disease is helpful for the long-term management of endometriosis. (P)** | 67(22.26) | 179(59.47) | 48(15.95) | 7(2.33) | / |
| **5. Believes that with proactive treatment and daily management, the disease can be overcome. (P)** | 68(22.59) | 179(59.47) | 52(17.28) | 2(0.66) | / |
| **6.** **Thinks that although the disease is difficult to prevent, it is necessary to reduce the risk of occurrence through regular check-ups, active exercise, etc. (P)** | 62(20.6) | 207(68.77) | 30(9.97) | 2(0.66) | / |
| **7.** **Feels that the hospital's educational efforts regarding endometriosis and long-term management are insufficient. (P)** | 8(2.66) | 60(19.93) | 160(53.16) | 68(22.59) | 5(1.66) |
| **8.** **Considers family understanding and encouragement crucial for boosting the patient's confidence in overcoming the disease and avoiding anxiety and unease. (P)** | 98(32.56) | 164(54.49) | 34(11.3) | 5(1.66) | / |
