# Supplementary material for: Patients’ knowledge, attitudes, and practices concerning endometriosis and its long-term management
Source: BMC Womens Health. 2025 Nov 28;25:633. doi: 10.1186/s12905-025-04187-z (PMC12750744; doi:10.1186/s12905-025-04187-z)
Supplement: Supplementary file 5 — Supplementary Material 5. [file 12905_2025_4187_MOESM5_ESM.docx]

**Table S4. Distribution of practice dimension responses**

| **Practice** | **Always** | **Often** | **Sometimes** | **Rarely** | **Never** |
| --- | --- | --- | --- | --- | --- |
| **1.** **Actively learning about the relevant knowledge of endometriosis and its long-term management. (P)** | 19(6.31) | 82(27.24) | 98(32.56) | 55(18.27) | 47(15.61) |
| **2.** **Paying attention to diet, consuming more fresh vegetables and fruits, salmon, and walnuts, and reducing the intake of high-fat meat. (P)** | 23(7.64) | 130(43.19) | 99(32.89) | 39(12.96) | 10(3.32) |
| **3.** **Making efforts to adjust emotions and actively dealing with the physical and emotional challenges that endometriosis may bring. (P)** | 21(6.98) | 138(45.85) | 104(34.55) | 29(9.63) | 9(2.99) |
| **4.** **Undergoing regular check-ups and follow-up appointments. (P)** | 64(21.26) | 151(50.17) | 55(18.27) | 27(8.97) | 4(1.33) |
| **5.** **Engaging in regular physical exercise. (P)** | 16(5.32) | 110(36.54) | 110(36.54) | 59(19.6) | 6(1.99) |
| **6.** **Actively participating in any educational activities organized by medical institutions regarding endometriosis and long-term management. (P)** | 3(1) | 25(8.31) | 61(20.27) | 112(37.21) | 100(33.22) |
| **7.** **Following medical advice for medication treatment. (P)** | 124(41.2) | 80(26.58) | 29(9.63) | 23(7.64) | 45(14.95) |
