# Supplementary material for: Patients’ knowledge, attitudes, and practices concerning endometriosis and its long-term management
Source: BMC Womens Health. 2025 Nov 28;25:633. doi: 10.1186/s12905-025-04187-z (PMC12750744; doi:10.1186/s12905-025-04187-z)
Supplement: Supplementary file 6 — Supplementary Material 6. [file 12905_2025_4187_MOESM6_ESM.docx]

Table S5. Spearman correlation analysis

|  | Age (years) | Education | Ethnicity | Employment | Monthly Household Income, Yuan | Marital status | Have children | Smoking | Drinking | Health insurance | Duration of endometriosis diagnosis | Have female relatives with endometriosis |
| --- | --- | --- | --- | --- | --- | --- | --- | --- | --- | --- | --- | --- |
| Age (years) | 1 |  |  |  |  |  |  |  |  |  |  |  |
| Education | -0.256 (P<0.001) | 1 |  |  |  |  |  |  |  |  |  |  |
| Ethnicity | 0.047 (P=0.418) | 0.072 (P=0.210) | 1 |  |  |  |  |  |  |  |  |  |
| Employment | -0.067 (P=0.246) | -0.224 (P<0.001) | 0.111 (P=0.055) | 1 |  |  |  |  |  |  |  |  |
| Monthly Household Income, Yuan | -0.153 (P=0.008) | 0.052 ()P=0.369) | -0.045 (P=0.436) | -0.048 (P=0.408) | 1 |  |  |  |  |  |  |  |
| Marital status | 0.464 (P<0.001) | -0.252 (P<0.001) | 0.018 (P=0.749) | -0.131 (P=0.023) | 0.001 (P=0.990) | 1 |  |  |  |  |  |  |
| Have children | -0.626 (P<0.001) | 0.258 (P<0.001) | 0.119 (P=0.039) | 0.029 (P=0.614) | 0.097 (P=0.093) | -0.481 (P<0.001) | 1 |  |  |  |  |  |
| Smoking | -0.116 (P=0.044) | -0.026 (P=0.648) | -0.017 (P=0.770) | -0.08 (P=0.167) | -0.023 (0.697) | -0.157 (P=0.006) | -0.038 (P=0.512) | 1 |  |  |  |  |
| Drinking | 0.167 (P=0.004) | -0.082 (P=0.154) | 0.043 (P=0.456) | 0.091 (P=0.113) | -0.446 (P<0.001) | 0.074 (P=0.200) | -0.094 (P=0.102) | 0.007 (P=0.898) | 1 |  |  |  |
| Health insurance | 0.167 (P=0.004) | 0.026 (P=0.655) | -0.028 (P=0.633) | 0.251 (P<0.001) | 0.160 (P=0.005) | -0.095 (P=0.101) | 0.063 (P=0.277) | 0.101 (P=0.080) | -0.013 (P=0.826) | 1 |  |  |
| Duration of endometriosis diagnosis | 0.219 (P<0.001) | 0.013 (P=0.817) | -0.103 (P=0.074) | -0.131 (P=0.023) | -0.053 (P=0.360) | 0.199 (P=0.001) | -0.184 (P=0.001) | -0.103 (P=0.074) | 0.057 (P=0.328) | -0.018 (P=0.760) | 1 |  |
| Have female relatives with endometriosis | 0.106 (P=0.065) | -0.146 (P=0.011) | -0.046 (P=0.425) | 0.015 (P=0.802) | -0.042 (P=0.466) | 0.049 (P=0.396) | -0.065 (P=0.258) | -0.049 (P=0.399) | -0.025 (P=0.662) | 0.02 (P=0.736) | -0.287 (P<0.001) | 1 |
